# Protective Effect of Phenolic Compounds Isolated from Mugwort (*Artemisia argyi*) against Contrast-Induced Apoptosis in Kidney Epithelium Cell Line LLC-PK1

**DOI:** 10.3390/molecules24010195

**Published:** 2019-01-07

**Authors:** Kem Ok Kim, Dahae Lee, Nguyen Tuan Hiep, Ji Hoon Song, Hae-Jeung Lee, Dongho Lee, Ki Sung Kang

**Affiliations:** 1 Department of Biosystems and Biotechnology, College of Life Science and Biotechnology, Korea University, Seoul 02841, Korea; kikeko520@gmail.com (K.O.K.); nguyentuanhiep.2710@gmail.com (N.T.H.); 2 School of Pharmacy, Sungkyunkwan University, Suwon 440-746, Korea; pjsldh@naver.com; 3 College of Korean Medicine, Gachon University, Seongnam 13120, Korea; jhsong@gachon.ac.kr; 4 Department of Food and Nutrition, College of Bio-Nano technology, Gachon University, Seongnam 13120, Korea; skysea@gachon.ac.kr

**Keywords:** contrast agent, iodixanol, cytotoxicity, MAPKs, caspase-3, kidney injury molecule-1

## Abstract

We investigated whether 14 phenolic compounds isolated from *Artemisia argyi* could prevent the apoptotic damage caused by iodixanol, an iodinated contrast agent, on LLC-PK1 cells. Iodixanol was used to induce cytotoxicity in LLC-PK1 cells. Apoptotic cell death was observed as the fluorescence intensity emitted by annexin V and Hoechst 33342 stains. Western blotting was used to detect specific proteins. Seven phenolic compounds protected against iodixanol-induced LLC-PK1 cell death in a concentration-dependent manner. Among them, methyl caffeate exerted the strongest protective effect, and co-treatment with 50 and 100 μM methyl caffeate decreased intracellular reactive oxygen species elevated by 25 mg/mL iodixanol. In addition, the treatment of LLC-PK1 cells with iodixanol resulted in an increase in apoptotic cell death, which decreased by co-treatment with methyl caffeate. Iodixanol caused a cytotoxicity-related increase in the phosphorylation of extracellular-signal-regulated kinase, c-Jun N-terminal kinase, and P38; and a similar increase in the expression levels of kidney injury molecule-1 and cleaved caspase-3. However, the up-regulation of these proteins was reversed by co-treatment with methyl caffeate. These findings suggest that phenolic compounds isolated from *A. argyi* play an important role in protecting kidney epithelium cells against apoptotic damage caused by iodixanol.

## 1. Introduction

Contrast agents are widely used to improve the visibility of blood vessels and internal organs, including the tissues and urinary tract in patients undergoing elective coronary procedures [1]. Based on osmolality, contrast agents are classified into three distinct groups—nonionic low-osmolar iopromide, ionic ioxitalamate, and isoosmolar iodixanol [1,2]. Although newer and safer contrast agents have been developed, several recent studies have shown that the incidence of renal dysfunction persists after contrast agents use [3,4,5].

Contrast agent-induced toxicity in renal proximal tubular cells has been implicated in the pathogenesis of acute kidney injury [4]. The cytotoxicity of contrast agents towards renal proximal tubular cells was indicated in in vitro and in vivo researches as well as in clinical trials [6,7]. The major reasons for cytotoxicity are the reduction in medullary blood flow and direct renal proximal tubular cell damage. The latter is related to the hypoxia-mediated formation of reactive oxygen species (ROS) [2,8,9]. Therefore, the prevention of the formation of ROS can be beneficial in protecting against contrast agent-induced renal proximal tubular cell damage. Many studies have demonstrated that *N*-acetylcysteine (NAC), a known antioxidant, prevents contrast agent-induced nephrotoxicity in the human embryonic kidney cell line, the porcine renal proximal tubular cell line, and the canine Madin-Darby distal tubular renal cell line [1,8,10]. Vitamin E also protects against contrast agent-induced nephrotoxicity in patients undergoing elective coronary procedures [11,12].

*Artemisia argyi*, an aromatic herb belonging to the *Asteraceae* family, has been mainly used in traditional oriental medicine for treating various diseases, such as menstrual disorders, infertility, uterine bleeding, and inflammatory diseases [13,14,15,16,17]. In addition, the extract of *A. argyi* is known to reduce ethanol-induced gastrointestinal damage [18], carbon tetrachloride (CCl_4_)-induced hepatic damage [19], and cerulein-induced pancreatic damage [20] in rats. *A. argyi* contains phytochemicals such as sesquiterpenoids [21,22,23], triterpenoids [24,25], phenolics, and flavonoids [15,26,27,28]. These phytochemicals, isolated from *A. argyi,* exhibit various biological and pharmaceutical activities, including anti-oxidant, anti-inflammatory and anti-apoptosis activities [15,22,23,28]. In our previous study, we showed that artemetin, a flavonoid isolated from *A. argyi*, can protect against contrast agent-induced cytotoxicity in renal proximal tubular cells through the inhibition of ROS generation and apoptosis [28]. Since *A. argyi* contains a large number of compounds that possess biological activity [13], we continued our efforts to find more active compounds from *A. argyi* that exert protective effects against contrast agent-induced toxicity in renal proximal tubular cells and hypothesized that flavonoids may attenuate contrast agent-induced cytotoxicity in renal proximal tubular LLC-PK1 cells and focused on elucidating the molecular mechanism involved.

## 2. Results

### 2.1. Protective Effect of Phenolic Compounds Isolated from A. argyi on Iodixanol-Induced Renal Proximal Tubular LLC-PK1 Cell Death 

**Figure 1:**
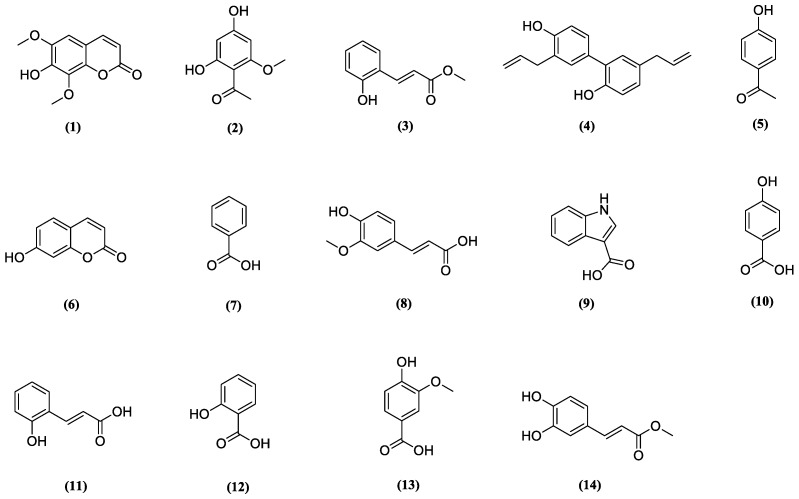
Chemical structures of phenolic compounds 1–14 identified from *Artemisia argyi*. Isofraxidin (**1**), 4,6-dihydroxy-2-methoxyacetophenone (**2**), methyl 3-(2-hydroxyphenyl)acrylate (**3**), honokiol (**4**), 4-hydroxyacetophenone (**5**), umbelliferone (**6**), benzoic acid (**7**), trans-ferulic acid (**8**), 1H-indole-3-carboxylic acid (**9**), 4-hydroxybenzoic acid (**10**), 3-(2-hydroxyphenyl)acrylic acid (**11**), salicylic acid (**12**), vanillic acid (**13**) and methyl caffeate (**14**).

**Figure 2:**
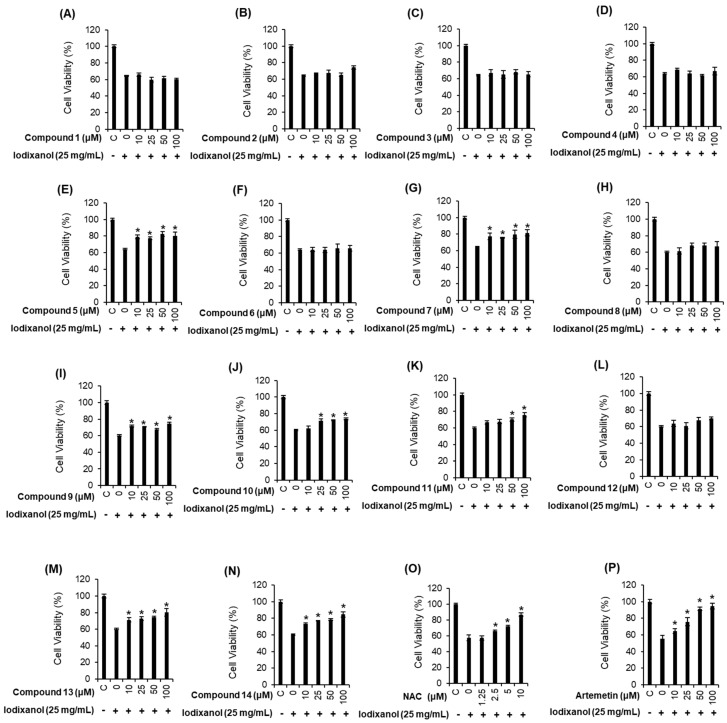
Comparison in the protective effects of phenolic compounds isolated from *Artemisia argyi* (**A**–**N**), *N*-acetylcysteine (NAC, positive control) (**O**) and artemetin (**P**) on the iodixanol-induced cytotoxicity in LLC-PK1 cells. Isofraxidin (**1**), 4,6-dihydroxy-2-methoxyacetophenone (**2**), methyl 3-(2-hydroxyphenyl)acrylate (**3**), honokiol (**4**), 4-hydroxyacetophenone (**5**), umbelliferone (**6**), benzoic acid (**7**), trans-ferulic acid (**8**), 1H-indole-3-carboxylic acid (**9**), 4-hydroxybenzoic acid (**10**), 3-(2-hydroxyphenyl)acrylic acid (**11**), salicylic acid (**12**), vanillic acid (**13**) and methyl caffeate (**14**). Control cells were treated with the vehicle only (mean ± SD, *n* = 3, * *p* < 0.05 compared to the iodixanol-treated group).

To evaluate the protective effect of 14 phenolic compounds isolated from *A. argyi* (Figure 1) on iodixanol-induced renal proximal tubular LLC-PK1 cell death, the cells were treated with phenolic compounds and NAC as a positive control for 2 h, and then further treated with 25 mg/mL iodixanol for 3 h. As shown in Figure 2A, treatment of 25 mg/mL iodixanol decreased the viability of the cells to 64.35 ± 0.71% compared to that of the control cells. The reduction in LLC-PK1 cell viability in response to iodixanol-induced damage recovered to 80.1 ± 4.5%, 81.4 ± 3.6%, 84.5 ± 3.0% and 86.1 ± 2.2% with the co-treatment of 100 μM of compounds 5, 7, 13, and 14, respectively (Figure 2E,G,M,N). Although the protective effects of these four compounds were similar, compound 14 (methyl caffeate) showed the strongest effect (Figure 2N). The effect of methyl caffeate was similar to that of the recovered cell viability of 86.9 ± 2.6% with 10 mM NAC (Figure 2O) and 25 μM artemetin (Figure 2P), which is a flavonoid compound that has a protective effect against iodixanol [28]. Therefore, further mechanism studies were carried out with methyl caffeate, since it represented sufficient protection against cell death caused by iodixanol.

### 2.2. Effect of Methyl Caffeate on Iodixanol-Induced Apoptosis and ROS Generation in LLC-PK1 Cells

**Figure 3:**
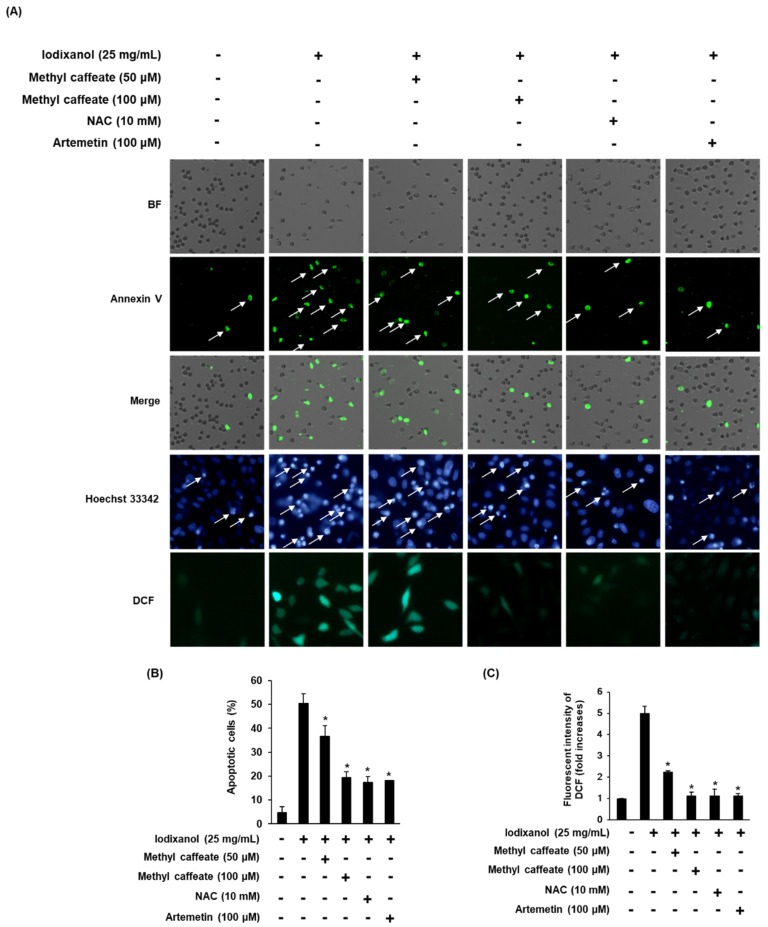
Effects of methyl caffeate isolated from *Artemisia argyi*, NAC and artemetin on apoptosis in LLC-PK1 cells exposed to 25 mg/mL of iodixanol scored by an image-based cytometric assay, Hoechst 33342 staining and DCFH-DA staining. (**A**) Representative images of apoptosis detection and intracellular reactive oxygen species (ROS) levels. (**B**) Percentage of Annexin V stained apoptotic cells. (**C**) Fluorescence intensity of 2′, 7′- dichlorodihydrofluorescein (DCF). Control cells were treated with the vehicle only (mean ± SD, *n* = 3, * *p* < 0.05 compared to the iodixanol-treated group).

We tested whether methyl caffeate could reduce iodixanol-induced apoptosis in LLC-PK1 cells. Treatment of 25 mg/mL iodixanol increased the fluorescence intensity of Hoechst 33342 in cells. In contrast, the treatment of 50 and 100 μM methyl caffeate and 10 mM NAC significantly reduced the iodixanol-induced increase in fluorescence intensity of Hoechst 33342 (Figure 3A). Similarly, the percentage of apoptotic cells with annexin V conjugated with V Alexa Fluor 488 (green fluorescence) increased significantly by 50.33 ± 4.16% by treatment with 25 mg/mL iodixanol, whereas the corresponding fluorescence was decreased by the treatments of 50 and 100 μM methyl caffeate and 10 mM NAC to 36.66 ± 4.50%, 19.33 ± 2.51% and 17.33 ± 2.51%, respectively (Figure 3B). We then explored whether methyl caffeate could decrease iodixanol-induced ROS generation in LLC-PK1 cells. Fluorescence intensity of 2′,7′- dichlorodihydrofluorescein (DCF) (in terms of fold increase) was significantly increased by 5.00 ± 0.32-fold by treatment with 25 mg/mL iodixanol, whereas it decreased by 2.25 ± 0.05-, 1.315 ± 0.16-, and 1.15 ± 0.27-fold by treatment with 50, 100 μM methyl caffeate and 10 mM NAC, respectively (Figure 3C).

### 2.3. Effect of Methyl Caffeate on Expression Levels of MAP Kinase (JNK, ERK and P38), Kidney Injury Molecule-1 (KIM-1) and Cleaved Caspase-3 on Iodixanol-Treated LLC-PK1 Cells

**Figure 4:**
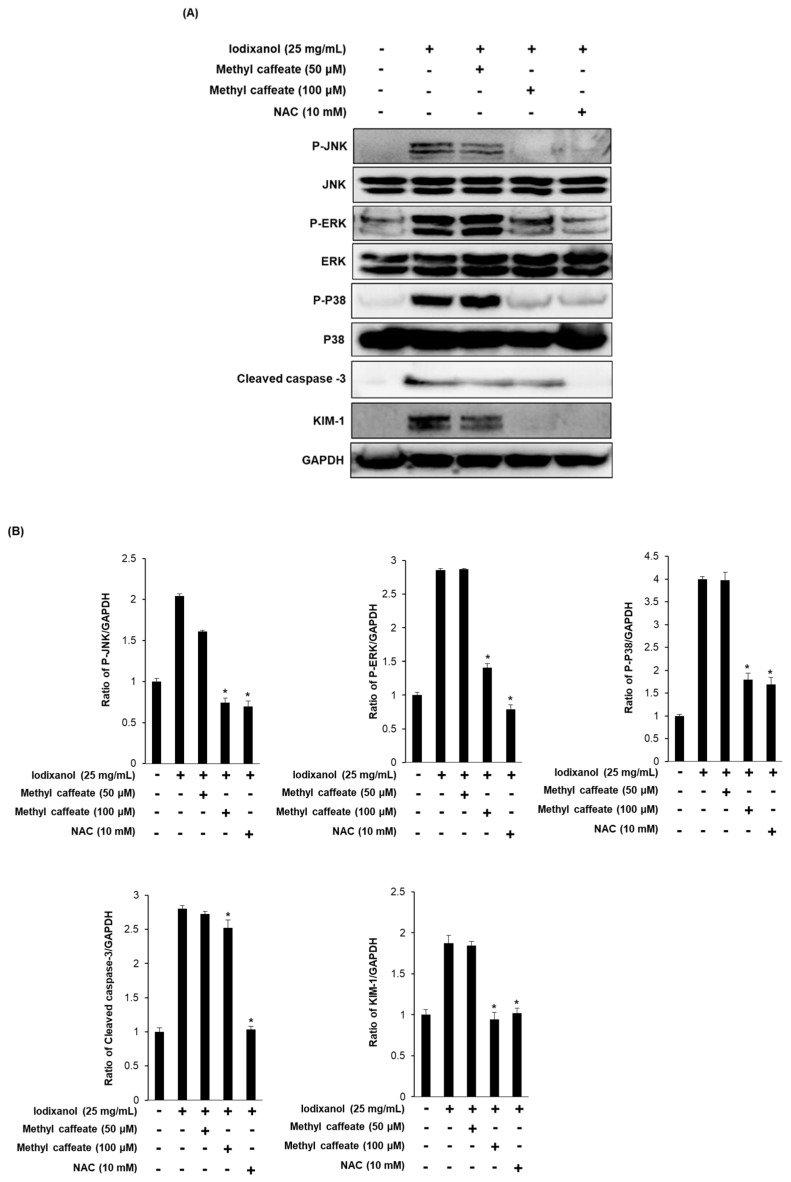
Effect of methyl caffeate on MAPKs, cleaved caspase-3 and kidney injury molecule-1 (KIM-1) in LLC-PK1 cells. (**A**) Effect of methyl caffeate on expression levels of MAPKs (JNK, P38 and ERK), cleaved caspase-3 and KIM-1 in LLC-PK1 cells exposed to 25 mg/mL iodixanol by Western blotting. (**B**) Each bar presents the densitometric quantification of Western blot bands. Control cells were treated with the vehicle only (mean ± SD, *n* = 3, * *p* < 0.05 compared to the iodixanol-treated group).

To explore the molecular mechanism of the protective effect of methyl caffeate, cells were exposed to 25 mg/mL iodixanol in the presence or absence of compound 14 and NAC, followed by Western blot analysis to evaluate the phosphorylation of ERK, JNK, and P38 and the expression levels of KIM-1 and cleaved caspase-3. Exposure of LLC-PK1 cells to 25 mg/mL iodixanol resulted in changes in the phosphorylation of MAPKs (ERK, JNK, and P38), whereas methyl caffeate significantly decreased the iodixanol-induced phosphorylation of MAPKs to control levels (Figure 4A). In addition, the elevated protein expression levels of KIM-1 and cleaved caspase-3 were decreased after treatment with 100 μM methyl caffeate (Figure 4A). This inhibitory effect of methyl caffeate on expressions of apoptosis-related proteins was similar to that of the 10 mM NAC, a positive control group (Figure 4B).

## 3. Discussion

In this study, we investigated the effects of 14 phenolic compounds isolated from *A. argyi* on iodixanol-induced cytotoxicity in LLC-PK1 cells. LLC-PK1 cells were chosen as the renal epithelial model system because previous studies found direct evidence for the toxicity of contrast agents during in vitro experiments using renal proximal tubular cells. This avoids any confounding variables, such as a variety of systemic physiological factors, that an in vivo system is subject to [2,5,29]. Moreover, contrast agents are concentrated in the renal proximal tubules and their concentration by water tubular reabsorption is much higher than in the plasma [1,2,30]. 

Among the 14 phenolic compounds isolated from *A. argyi*, 4-hydroxyacetophenone (5), benzoic acid (7), vanillic acid (13), and methyl caffeate (14) exhibited potent protective effects on iodixanol-induced LLC-PK1 cell death. The protective effects of these compounds were stronger than NAC, which is well-known an effective antioxidant. In previous studies on these four compounds, 4-hydroxyacetophenone isolated from *Tagetes mendocina* [31], vanillic acid isolated from *Origanum vulgare* [32], and methyl caffeate isolated from rice bran exhibited a scavenging activity on the DPPH radical [33]. Benzoic acid isolated from *Triticum aestivum* exhibited improving activities of superoxide dismutase (SOD) [34]. In addition, the intracellular ROS level in Madin Darby distal nonhuman tubular epithelial cells was increased by treatment with iodixanol (50, 100, 200 mg/mL) in a concentration-dependent manner, whereas it was decreased by pretreatment with 100 mM NAC [5]. In the present study, among the tested phenolic compounds, methyl caffeate proved to be the most effective phenolic compound of *A. argyi* and exhibited intracellular ROS scavenging activity, reflected by the decrease in oxidation of DCFH2 to DCF by intracellular ROS caused by iodixanol. In addition, methyl caffeate reduced the level apoptotic cell death caused by iodixanol. These results suggest that methyl caffeate may protect LLC-PK1 cells against oxidative damage and apoptotic cell death caused by iodixanol.

Previous studies suggest that contrast agent-induced renal damage is caused by molecular mechanisms related to oxidative stress, MAPKs and apoptosis [2,4,5,35]. MAPKs (JNK, P38 and ERK) were activated by the increased production of ROS, which then activated caspase-9 and caspase-3, leading to DNA fragmentation [2,5]. The phosphorylation of JNK and the expression of caspase-3 were increased in epithelial tubular cells collected from patients receiving 1 mL/kg iodixanol per hour. In an in vitro experiment, 100 mM NAC decreased the phosphorylation of P38 and JNK and the expression of caspase-3 in Madin Darby distal nonhuman tubular epithelial cells treated with 200 mg/mL iodixanol [5]. In addition, 100 mM NAC and 0.2 μg statins (known as HMG-CoA reductase inhibitors) decreased the phosphorylation of JNK and the expression of caspase-3 that were increased in both human embryonic proximal tubules cells and Madin Darby distal nonhuman tubular epithelial cells treated with 200 mg/mL iodixanol [35]. In previous clinical trials, KIM-1 was also an important molecule in contrast agent-induced renal damage and was activated in renal proximal tubules during ischemia and renal injury. The levels of urinary KIM-1 increased in urine collected from patients with contrast agent-induced nephropathy [36,37,38]. However, there is very limited information on KIM-1 expression in renal proximal tubular cells exposed to contrast agents. Thus, we evaluated the change in KIM-1 expression in renal proximal tubular cells exposed to contrast media, as well as the effect of antioxidants on KIM-1 expression. 

In this study, treatment with iodixanol significantly increased the phosphorylation of JNK, ERK and P38 and the expression levels of cleaved caspase-3 and KIM-1; these effects were reversed by treatment with methyl caffeate. These results suggest that the antioxidative effect of methyl caffeate may inhibit the phosphorylation of JNK, ERK and P38, which are activated in response to oxidative stress [39,40]; and inhibit, as well, the expression of caspase-3, which is activated both by extrinsic and intrinsic apoptosis pathways [41] and KIM-1.

## 4. Materials and Methods

### 4.1. Chemicals and Reagents

Ez-Cytox cell viability assay kit was purchased form Dail Lab Service Co. (Seoul, Korea). Iodixanol, 2′,7′- dichlorodihydrofluorescein diacetate (DCFH-DA) and Hoechst 33342 were purchased from Sigma–Aldrich (Saint Louis, MO, USA). BS was purchased from Invitrogen Co. (Grand Island, NY, USA). Dulbecco Modified Eagle Medium (DMEM) and Pierce™ BCA Protein Assay Kit were purchased from Thermo Scientific (Manassas, VA, USA). ECL Advance Western Blotting Detection Reagents were purchased from GE Healthcare (Little Chalfont, Buckinghamshire, UK). Tali image-based cytometric assay kit was purchased from Invitrogen (Temecula, CA, USA). Antibodies for c-Jun-N-terminal kinase (JNK), phospho-JNK, p44/42 MAP kinase (ERK), phospho-ERK, P38 mitogen-activated protein kinases (MAPKs), phospho-P38, kidney injury molecule-1 (KIM-1), glyceraldehyde 3-phosphate dehydrogenase (GAPDH), cleaved caspase-3 were purchased from Cell Signaling (Boston, MA, USA).

### 4.2. General Analytical Procedures

Thin-layer chromatography was carried out using pre-coated silica Q-gel 60 F254 plates (0.25 mm, Merck). Column chromatography (CC) was conducted using silica gel (Kieselgel 60, 230–400 mesh, Merck), and Sephadex LH-20 (18–111 µm, GE Healthcare AB, Stockholm, Sweden). HPLC was performed using the Varian Prostar 210 system. A YMC-Pack ODS-A column (5 μm, 250 × 20 mm i.d., YMC, Kyoto, Japan) was used for preparative HPLC analysis with an 8 mL/min flow rate. ESI-MS was performed on an LCQ Fleet Ion Trap mass spectrometer (Thermo Scientific, Madison, WI, USA). NMR spectral data were acquired by using a Varian 500-MHz NMR spectrometer (Inova 500 Spectrometer, Varian, Palo Alto, CA, USA). Tetramethylsilane was used as an internal standard, and chemical shifts data were expressed as δ value.

### 4.3. Plant Material

The dried leaves of *Artemisia argyi,* H. Lév., and Vaniot were obtained in April 2013 from the Gyeongdong herbal medicine market located in Seoul (Republic of Korea), and was identified by Dr. Je-Hyun Lee (College of Oriental Medicine, Dongguk University, Gyeongju, Republic of Korea). A voucher specimen of *A. argyi* (AA1-103-130429) was deposited at the Department of Biosystems and Biotechnology, Korea University (Seoul, Republic of Korea).

### 4.4. Extraction and Isolation of Phenolic Compounds from A. argyi

The leaves of *A. argyi* (3 kg) were extracted 3 times with 100% MeOH (18 L, 9 L, 9 L) at room temperature. The MeOH extract (420 g) was prepared after removing the organic solvent in vacuo, and dissolved with water (3.9 L). Sequentially, the aqueous solution was fractionated with EtOAc (3 × 1.3 L) and *n*-hexane (3 × 1.3 L) to produce dried EtOAc (78 g)- and *n*-hexane (50 g)-soluble extracts. The EtOAc fraction was subjected to silica gel CC and eluted with a gradient condition of CHCl_3_–MeOH (1:0 to 1:1) to give 9 sub-fractions designated as KO1-107-1 to -9. Sub-fraction KO1-107-5 (25 g) was further fractionated by silica gel CC and eluted with a gradient of CHCl_3_–acetone (1:0 to 1:1) to produce 8 fractions designated as KO1-120-1 to -8. The fraction KO1-120-3 (4.1 g) was subjected to Sephadex LH-20 CC and eluted with CHCl_3_–MeOH (1:1) to yield 11 fractions (designated as KO1-122-1 to -11). KO1-122-7 (519 mg) was fractionated by silica gel CC with a gradient elution condition of *n*-hexane–EtOAc (1:0 to 1:1) to yield 6 fractions designated as TH5-63-1 to -6. Fraction TH5-63-6 (73.3 mg) was separated by preparative HPLC (60% MeOH) to yield isofraxidin (**1**, 5.4 mg) [42]. Fraction KO1-122-10 (40.5 mg) was purified by preparative HPLC (35–100% MeOH in H_2_O) to produce 4,6-dihydroxy-2-methoxyacetophenone (**2**, 2.5 mg) [43], methyl 3-(2-hydroxyphenyl)acrylate (**3**, 1.9 mg) [44], and honokiol (**4**, 4.7 mg) [45]. KO1-120-4 (5.2 g) was separated by silica gel CC on Sephadex LH-20 and eluted with CHCl_3_–MeOH (1:1) to produce 8 fractions (designated as KO1-124-1 to -8). Fraction KO1-124-8 (186 mg) was purified by preparative HPLC (35–75% MeOH) to produce 4-hydroxyacetophenone (**5**, 6.5 mg) [46], umbelliferone (**6**, 9.0 mg) [47] and benzoic acid (**7**, 4.9 mg) [48]. KO1-107-6 (6.13 g) was subjected to Sephadex LH-20 CC and eluted with CHCl_3_–MeOH (1:1) to yield 7 fractions (designated as TH5-47-1 to -7). TH5-47-6 (125.9 mg) was separated by preparative HPLC (40–70% MeOH) to produce trans-ferulic acid (**8**, 6.7 mg) [49], vanillic acid (**13**, 14.8 mg) [50] and methyl caffeate (**14**, 1.5 mg) [51]. Fraction KO1-107-7 (7.1 g) was subjected to Sephadex LH-20 CC and eluted with CHCl_3_–MeOH 2:1) to produce 5 fractions (designated as TH5-57-1 to -5). Fraction TH5-57-4 (377.8 mg) was fractionated by silica gel CC and eluted with a gradient condition of *n*-hexane–EtOAc (1:0 to 1:1) to yield 7 fractions designated as TH5-61-1 to -7. TH5-61-1 (138.1 mg) was separated by preparative HPLC (35–80% MeOH) to produce 4-hydroxybenzoic acid (**10**, 17.1 mg) [50], 3-(2-hydroxyphenyl)acrylic acid (**11**, 70 mg) [52], and salicylic acid (**12**, 10.9 mg) [53]. TH5-57-5 (50.9 mg) was separated by preparative HPLC (25–85% MeOH) to produce 1H-indole-3-carboxylic acid (**9**, 4.1 mg) [54]. The structure of the 14 phenolic compounds isolated from *A. argyi* was characterized by NMR and ESI-MS analyses.

### 4.5. Cell Culture

LLC-PK1 cells, a porcine renal proximal tubular cell line, were purchased from the American Type Culture Collection (Rockville, MD, USA). The cells were then cultured in DMEM (Cellgro, Manassas, VA, USA), supplemented with 4 mM L-glutamine, 1% penicillin/streptomycin and 10% FBS (Invitrogen Co., Grand Island, NY, USA) in an atmosphere of 5% CO_2_ at 37 °C.

### 4.6. Quantification of Cellular Viability with Ez-Cytox Assay

We implemented methods used to study protective effects on iodixanol-induced cytotoxicity in kidney cells [28,55]. The cells were seeded at 1 × 10^4^ cells per well in 96-well culture plates and incubated for 24 h to promote adhesion. Then, the cells were either treated with 0.5% DMSO (control), or the indicated concentrations of flavonoid compounds. NAC treatment was used as a positive control (10 mM). After incubation for further 2 h, iodixanol (25 mg/mL) was added to each well and incubated for 3 h. The experiments were performed in triplicate and readings of the cell viability using the the Ez-Cytox assay kit according to the manufacturer’s instructions, with the absorbance value at 450 nm measured using a microplate reader (PowerWave XS; Bio-Tek Instruments, Winooski, VT, USA).

### 4.7. Quantification of DNA Fragmentation by Hoechst 33342 Staining

LLC-PK1 cells seeded at 4 × 10^5^ cells per well in 6-well plates were treated with control (0.5% DMSO), or compound 14 and NAC as the positive control compound (10 mM). After incubation for 2 h, iodixanol (25 mg/mL) was then added to each well and incubated for 3 h, followed by the addition of 2 μL of Hoechst 33342 solution and incubation for 10 min at 37 °C [56]. The stained cells were observed under an IX50 fluorescent microscope equipped with a CCD camera (Olympus, Tokyo, Japan).

### 4.8. Quantification of ROS Level by DCFH-DA Staining

LLC-PK1 cells seeded at 4 × 10^5^ cells per well in 6-well plates were treated with 0.5% DMSO (control), or compound 14, and 10 mM NAC (positive control compound). After incubation for 2 h, iodixanol (25 mg/mL) was added to each well and incubated for 3 h. Next, cells were incubated with 10 µM DCFH-DA for 30 min at 37 °C and then washed with phosphate-buffered saline (PBS). The stained cells were observed under an IX50 fluorescent microscope equipped with a CCD camera (Olympus, Tokyo, Japan) and the fluorescence intensity of DCF was measured at 495/517 nm (ex/em) using a SPARK 10M fluorescent mAcroplate reader (Tecan, Männedorf, Switzerland) [57].

### 4.9. Quantification of Apoptosis with Image-Based Cytometric Assay

LLC-PK1 cells seeded at 4 × 10^5^ cells per well in 6-well plates were treated with 0.5% DMSO (control), or compound 14, and 10 mM NAC (positive control compound). After incubation for 2 h, iodixanol (25 mg/mL) was added to each well and incubated for further 3 h. Next, cells were harvested and washed with PBS. The cells were resuspended at 5 × 10^5^ cell/100 μL in binding buffer and an aliquot of 100 μL was incubated with 5 μL of annexin V Alexa Fluor 488 for 30 min in the dark at room temperature. The Annexin V-positive-stained cells were measured by a Tali image-based cytometer (Invitrogen, Temecula, CA, USA) [58].

### 4.10. Western Blotting Analysis

LLC-PK1 cells seeded at 4 × 10^5^ cells per well in 6-well plates were treated with 0.5% DMSO (control), or compound 14, and 10 mM NAC (positive control compound). Protein samples were electrophoresed, transferred, and detected by epitope-specific primary antibodies to JNK (#9252), P-JNK (#9251), ERK (#4695), P-ERK (#4370), P38 (#8690), P-P38 (#4511), KIM-1 (#14971), cleaved caspase-3 (#9661), GAPDH (#2118), and HRP conjugated anti-rabbit (#7074) antibodies as reported previously [59].

### 4.11. Statistical Analysis

Statistical analysis was conducted using analysis of variance (ANOVA) followed by a multiple comparison test with a Bonferroni adjustment. The analysis was carried out using SPSS ver. 19.0 (SPSS Inc., Chicago, IL, USA). All the assays were done in triplicate for each assay and were repeated at least three times. The data with P values of less than 0.05 were considered as a statistically significant effect.

## 5. Conclusions

Our findings indicate that methyl caffeate, a phenolic compound isolated from *A. argyi*, protects against iodixanol-induced LLC-PK1 cell death, an action that we showed to be due to the combination of antioxidant and antiapoptotic effects resulting from the inhibition of the activities of MAPKs (JNK, P38 and ERK), caspase-3, and KIM-1. These findings suggest the potential of methyl caffeate as a new and efficient therapeutic approach to prevent contrast agent-induced cytotoxicity and preserve renal function during the pathogenesis of an acute kidney injury.
